# Development of a Culinary Medicine Curriculum to Support Nutrition Knowledge for Gastroenterology Fellows and Faculty

**DOI:** 10.3390/nu16030404

**Published:** 2024-01-30

**Authors:** Karen L. Lindsay, Jennifer Kennedy, Daniel Kim, Ankush Kalra, Nimisha K. Parekh

**Affiliations:** 1 Department of Pediatrics, School of Medicine, University of California Irvine, Orange, CA 92686, USA; 2 Susan Samueli Integrative Health Institute, Susan and Henry Samueli College of Health Sciences, University of California Irvine, Irvine, CA 92617, USA; 3 Division of Gastroenterology, Department of Medicine, School of Medicine, University of California Irvine, Orange, CA 92686, USA

**Keywords:** culinary medicine, nutrition education, gastroenterology, fellowship

## Abstract

Gastroenterologists encounter many nutrition-related disorders in their practice, yet the nutritional needs of patients with chronic gastrointestinal (GI) and liver disease are largely unaddressed by treating physicians, due to suboptimal nutrition education. To address this gap, we developed and piloted a culinary medicine course for a GI fellowship training program. The objective of this study is to describe the development, implementation, and acceptability of the course. A registered dietitian, a chef instructor, and a gastroenterology clinical professor trained in culinary medicine developed the four-class tailored curriculum and delivered the classes remotely. Each class had a theme related to commonly encountered GI disorders and included hands-on meal preparation, a nutrition lecture, and a patient case study discussion. Post-course feedback surveys were disseminated. Twenty-three GI physicians enrolled in the course and the attendance rates in classes 1–4 were 83%, 65%, 61%, and 48%, respectively. Among 15 completed feedback surveys, 80% reported that the class contents were either moderately or extremely useful and all endorsed the curriculum for other gastroenterologists. Future studies of culinary medicine programs tailored to medical specialties should identify strategies to maintain engagement and assess the impact on nutrition knowledge, competencies, and translation of these new skills to clinical practice.

## 1. Introduction

Gastroenterologists frequently treat diseases that are impacted by diet, including fatty liver disease, inflammatory bowel disease, celiac disease, irritable bowel syndrome, gastroesophageal reflux disease, and complications of obesity. Many gastroenterology fellowship training programs in the United States do not provide nutrition education due to a lack of faculty expertise and resources, such as a dedicated division dietician. Currently, nutrition education often focuses on vitamin and nutrient deficiencies, total parenteral nutrition, and nutrition in critically ill patients; these are the most tested nutrition topics in the gastroenterology board certification exam [1,2]. There are several online tools, books, and applications available to enhance the application of nutrition interventions for gastroenterologists and their patients [3], but these require self-learning and may not be actively sought out by practicing physicians. Meanwhile, nutrition education during gastrointestinal (GI) fellowships remains sparse and the nutritional needs of patients with chronic GI and liver disease are largely unaddressed by treating physicians. Studies have demonstrated patients’ preference for more specific focused dietary guidance to achieve improvements in dietary quality and confidence in food preparation through culinary interventions [4,5,6,7]. Physicians are often ill-equipped with infrastructure or evidence-based materials to provide more sophisticated counseling. The failure of gastroenterologists to effectively offer dietary treatments does a grave disservice to patients. 

Culinary medicine is an emerging discipline and training modality within clinical and public health education that provides medical trainees (e.g., medical students, nursing students, and dietetic interns), healthcare professionals, and community members with experiential food-based nutrition knowledge and the culinary skills needed for implementation [8,9]. Observational studies and randomized trials have shown that culinary medicine interventions based on Mediterranean diet principles lead to higher adherence to the Mediterranean diet and improved dietary counseling competencies among medical students, residents, physicians, and nurses [4,5,6,10,11].

While culinary medicine classes are traditionally taught in-person in a kitchen setting, virtual adaptations of culinary medicine have been successfully delivered in medical schools, with evidence of feasibility and a comparable impact to in-person classes [12,13,14]. We piloted a remotely delivered culinary medicine course for the GI fellowship training program at the University of California, Irvine (UCI). As gastroenterology faculty play a key role in mentoring fellows, we also included faculty in our course to help ensure that consistent evidence-based messaging on nutrition specific to the discipline was delivered to fellows. This paper describes the curriculum’s development, implementation, and acceptability. We also discuss recommendations for program scalability and opportunities for outcome evaluation within academic medical systems. 

## 2. Materials and Methods

In January–March 2021, the GI Culinary Medicine course was promoted to gastroenterology fellows and faculty at UCI at division meetings and via email from the last author, a Clinical Professor of Gastroenterology and the Associate Dean for Faculty Development in the UCI School of Medicine. The division at that time consisted of 39 individuals; 22 faculty, 14 fellows and 3 physician assistants. Fellows and faculty who volunteered to participate provided informed consent and completed a short baseline survey about their demographic information (age and racial identity) and whether they had any prior formal training in nutrition. The study was approved by the UCI Institutional Review Board.

### 2.1. Curriculum Development and Implementation

A registered dietitian, a chef, and a gastroenterologist with expertise in teaching and directing culinary medicine programs for medical students developed the course contents, utilizing the Health meets Food curriculum as a basis. The Health meets Food curriculum was developed by experts in lifestyle medicine, dietetics, and culinary education, to standardize the delivery of evidence-based culinary medicine courseware to healthcare professionals and trainees. The programming is used by over 60 academic medical centers across the United States and is designed to help physicians and other medical professionals understand and communicate the impact of good nutrition on their patients’ health. By marrying a curriculum based on basic science with clinical education and hands-on cooking classes, Health meets Food teaches medical trainees and physicians about the benefits of nutrition-related lifestyle changes and how to guide their patients towards healthier choices, by incorporating dietary intervention strategies into the practice of medicine [15,16].

Our GI culinary medicine program streamlined the broader Health meets Food curriculum to focus on gastroenterology-specific topics, including celiac disease, a low FODMAP diet (Fructo-, Oligo-, Di-, Monosaccharides and Polyols), an anti-inflammatory diet, and the Mediterranean diet, in four modules, as outlined in Table 1. In addition, an overview of macronutrients (fats, proteins, and carbohydrates), mindfulness, motivational interviewing, and the gut microbiome was integrated into the modules. Each module consisted of a pre-session online learning component using Health meets Food educational materials, a live hands-on cooking class led by a trained chef, and nutrition education and case study discussion led by a registered dietitian. Case studies included a diet recall and discussion about appropriate dietary counseling for the patient. Recipes were designed by the chef to align with the nutrition topic of each class and to demonstrate different types of culinary skills.

Due to COVID-19 restrictions, each 2 h class was conducted remotely through Zoom. Participants were provided with key ingredients for each recipe and basic cooking equipment for the virtual at-home cooking classes. The classes were held in spring 2021 during the evening, to accommodate physicians’ schedules. Attendance rates were recorded for each class.

### 2.2. Assessment of Course Acceptability

A de-identified post-course evaluation survey was administered to gain participant feedback on the perceived utility of (a) the nutrition lectures and (b) the hands-on cooking sessions for learning about the nutrition concepts and culinary skills for the GI topics covered. These questions were asked using a seven-point Likert scale, ranging from Extremely Useless to Extremely Useful, and responses were summarized descriptively and in a bar chart. The second survey item asked if they would recommend this tailored culinary medicine course to other gastroenterologists (the response options were yes/no/unsure). A final open-ended question solicited additional feedback from participants about their impression of the overall course.

## 3. Results

There were 23 total participants (representing 59% of the gastroenterology division), of whom 15 were gastroenterology fellows and 8 were gastroenterology faculty members. Most participants were between the ages of 30 and 39 (69%) while 22% were ≥40 years of age and 9% were 25–29 years of age. For racial identity, 61% of individuals identified as Asian, 26% as Caucasian, and 4% as Hispanic, with 9% listing themselves under Other. Data on participant sex were not recorded. None of the participants reported having previously received any formal nutrition training.

Attendance rate was high in class 1 (82.6%; *n* = 19) but was lower in subsequent classes, with *n* = 15 attending class 2 (65.2%), *n* = 14 in class 3 (61.0%), and *n* = 11 in class 4 (47.8%). Adherence rates at the participant level were not recorded.

**Figure 1:**
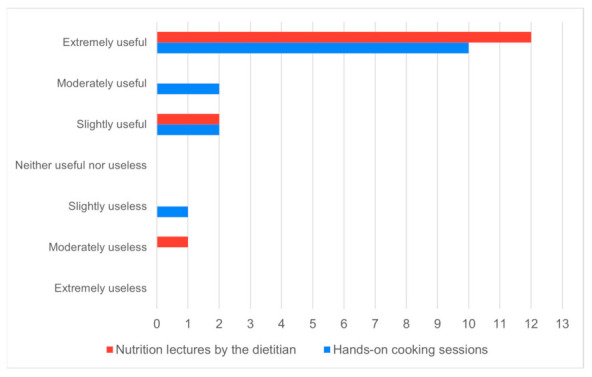
Perceived utility of nutrition lectures and hands-on cooking sessions for learning about nutrition concepts and culinary skills.

Fifteen participants responded to the post-course feedback survey (65% response rate). Of these, 80% reported that the nutrition lecture by the dietitian as well as the hands-on cooking sessions were either moderately or extremely useful (Figure 1). All respondents stated they would recommend this culinary medicine curriculum to other gastroenterologists. 

The following five open-ended feedback responses were provided by respondents to the feedback survey:

“Instructors were excellent and I learned a lot. More time for lectures would have been even better.”

“Chef was sometimes too fast to keep up with.”

“I always felt like I was behind Chef on timing of cooking, but in person this probably be more manageable!”

“Superb-fun-lively-educational and delicious!”

“Wonderful! Engaging and instructive”

## 4. Discussion

This study reports the successful delivery and acceptability of a tailored culinary medicine curriculum for fellows in gastroenterology. We achieved a high initial participation rate, although this reduced as the course progressed, and feedback received was highly positive. 

Nutrition education is an important component of empowering patients and improving clinical outcomes [17,18]. However, formal nutrition training is lacking in medical education [19,20,21], even in gastroenterology fellowship programs. None of the GI fellows or faculty involved in this study reported prior formal nutrition training. Most of the nutrition education that GI fellows receive focuses on malnutrition and total parenteral nutrition, yet most patients seen in GI clinics face very different issues, ranging from excess calorie consumption and obesity to the need for specialized dietary plans for common GI diseases. Our pilot culinary medicine curriculum was established to help fill this gap. 

Feedback survey responses from our GI culinary medicine program indicate the course was highly acceptable. Almost all participants who provided feedback perceived some benefit from both the didactic nutrition and hands-on culinary components of the curriculum. Open-ended feedback responses indicate a high level of enjoyment and desire for further nutrition education in this format. However, we acknowledge that feedback responses were likely biased towards those who were most engaged in the program. 

GI fellowship is time- and learning-intensive. Our culinary medicine curriculum utilized an interactive model for education as well as team-building and social interaction, factors that can bolster fellowship camaraderie. The curriculum was streamlined to provide focused GI education in an after-hours setting for the participants, so as not to impact other fellowship responsibilities. Despite this, the attendance rate dropped as the course progressed. Unfortunately, reasons for non-attendance or the characteristics of those who attended fewer, or no classes, were not recorded. Previous reports of culinary medicine programs delivered in medical schools primarily involved medical students as participants [12,22,23]. These studies report high adherence rates to the culinary medicine program, which is to be expected, as the classes are generally components of course electives and non-completion may impact grades. A paucity of studies report the implementation of culinary medicine programs among graduated medical trainees at the level of residents or fellows. Johnston et al. [24] described the delivery of a single culinary medicine class to Family Medicine residents and the self-reported impact on nutrition counseling confidence and competency, with beneficial effects identified. However, the single class format in that study did not allow for the longitudinal assessment of residents’ engagement and adherence to the culinary medicine program. 

To our knowledge, this pilot study is the first to describe a culinary medicine curriculum for physician fellows and the first tailored culinary medicine curriculum for the gastroenterology discipline. Limitations of the study include a small sample size, a failure to record participant-level adherence rate (i.e., it is unknown how many attended all four classes), and a failure to capture feedback data from all who attended at least one class, as well as reasons for non-participation and drop-out as the course progressed. Because the feedback surveys were most likely completed by those who attended most of the classes, we acknowledge there is likely to be some degree of response bias regarding acceptability of the program. However, we note that conflicting clinical duties were plausible reasons for non-attendance in classes, which does not necessarily imply low interest in the program (although specific reasons for non-attendance were not recorded for each participant). The lack of data on participant sex also limits the generalizability of the results. We also did not conduct any formal assessment of changes in physicians’ nutritional knowledge and competencies from pre- to post-course, or the translation of this knowledge gained into dietary counseling for their patients. Future efforts to implement a focused culinary medicine curriculum among trained physicians should invest in strategies to maintain engagement, such as offering continuing medical education credits, and should include an assessment of nutritional knowledge gained and dietary counseling confidence. It would also be important to assess how patients are benefiting indirectly from this nutrition curriculum by examining the impact on patient health outcomes. We propose that similar nutritional education programs can be implemented into various gastroenterology fellowships and outcomes assessed longitudinally.

Overall, this study demonstrates that a culinary medicine curriculum can be easily integrated into a GI fellowship and perceived as both beneficial and enjoyable as a method of nutrition education for GI fellows and faculty. It also demonstrates how virtual platforms for education can be an impactful way to deliver skills-based training to medical trainees. Future studies could assess the impact of culinary medicine classes on provider knowledge and skills, as well as nutritional knowledge translation to the patient care setting.

## Figures and Tables

**Table 1:** Overview of GI culinary medicine curriculum.

Class Number	Nutrition Concepts Taught	Recipes Prepared	Case Study Type
1	Busting fad diets; evidence for Mediterranean and plant-based dietary patterns.	Simple salad with balsamic vinaigrette; quinoa cooked three ways; chicken and veggie stir-fry	Hepatic steatosis
2	Anti-inflammatory diet; fiber and gut health.	Brown rice lentil pilaf; curried coconut chickpeas	Ulcerative colitis
3	Low FODMAP diet; mindfulness; motivational interviewing.	Tofu spring rolls with rice noodles and peanut dipping sauce; Thai spiced chicken and collard green rolls with brown rice	Irritable bowel syndrome
4	Gluten-free diet; sugar substitutes; gut microbiome.	Savory oat and quinoa porridge with poached egg; shakshuka; gluten-free granola	Celiac disease

## Data Availability

The data presented in this study are available on request from the corresponding author.
